# Nurse bees regulate the larval nutrition of developing workers (*Apis mellifera*) when feeding on various pollen types

**DOI:** 10.1093/jee/toae045

**Published:** 2024-04-12

**Authors:** Madlen Kratz, Robert Manning, Kenneth Dods, Boris Baer, Dominique Blache

**Affiliations:** School of Molecular Sciences, The University of Western Australia, Bayliss Building (M316), Crawley, WA 6009, Australia; School of Agriculture and Environment (M087), University of Western Australia Institute of Agriculture, The University of Western Australia, Crawley, WA 6009, Australia; New South Wales Department of Primary Industries, 815 Tocal Road, Paterson, NSW 2421, Australia; Formerly, Plant Biosecurity, Western Australian Department of Agriculture and Food, South Perth, WA 6151, Australia; RMO Consultancy, 301 Forrest Road, Bibra lake, WA, Australia; Formerly, ChemCentre, Resources and Chemistry Precinct, Bentley, WA, Australia; SAGE Consultancy, Perth, WA, Australia; Center for Integrative Bee Research (CIBER), Department of Entomology, The University of California, Riverside, CA 92506, USA; School of Agriculture and Environment (M087), University of Western Australia Institute of Agriculture, The University of Western Australia, Crawley, WA 6009, Australia

**Keywords:** amino acid, fatty acid, mineral, body composition, honey bee

## Abstract

Nutrition has been identified as a key driver of colony health and productivity. Yet, in honey bees, relatively little is known about how the vast variety of natural pollen sources impact larval development. The impact of the nutritional quality of 4 naturally occurring pollen sources, of importance to the Western Australian beekeeping industry, was tested on honey bee (*Apis mellifera* L.) development. Bee packages consisting of 800 g of bees and a mated sister queen were assigned to 40 nucleus hives and randomly allocated to one of the 4 feed treatments (10 colonies each) of marri (*Corymbia calophylla* Lindl.), jarrah (*Eucalyptus marginata* Sm.), clover (*Trifolium repens* L.), and canola (*Brassica napus* L.) pollen. Emerging bees were collected once the first bees started hatching on the assigned feed sources. Newly emerging bees were weighed individually, and body composition was measured in batches according to the feed treatment groups. Food consumption was recorded for the duration of the experiment. Nurse bees successfully raised young adult workers from the larval stage until emergence when fed with one of 4 pollen patties with different nutritional qualities. There was no difference in the body composition or weight of emerging bees fed on the different pollen types. However, the body weight of bees increased over time, most likely related to colony size and structure. With the type of pollen patties having little impact on larval development, the availability of pollen may be more important than its composition, providing bees have access to all essential nutrients.

## Introduction

Nutrition is crucial as it plays a key role beyond meeting the nutritional needs of an organism for survival. Globally, it is now understood that the diet bees have access to determines not only their longevity (Kleinschmidt and Kondos 1976, Brodschneider and Crailsheim 2010), but also the effects of parasites and susceptibility to disease (DeGrandi-Hoffman et al. 2010, Annoscia et al. 2017, Branchiccela et al. 2019), spring buildup and overwintering survival (Mattila and Otis 2006a, 2006b, Brodschneider and Crailsheim 2010, Branchiccela et al. 2019), ultimately affecting hive productivity and functioning (Farrar 1937, 1944, Kleinschmidt and Kondos 1976, 1978).

Honey bees (*Apis* spp.) regulate their nutritional requirements at the colony level (Herbert 1992), whereby nurse bees are pivotal to the nutrition of the colony. Nurse bees feed all colony members, from larvae to the queen, drones, and adult bees such as foragers (Haydak, 1970, Crailsheim, 1990, 1992, 1998). Pollen is the bees’ primary source of protein, fats, minerals, and vitamins. Nurse bees require a large amount of protein for the development of functional mandibular and hypopharyngeal glands (Herbert 1992, Brodschneider and Crailsheim 2010, Wright et al. 2018) and for the production of jelly (Crailsheim, 1992).

Adequate quantity and quality of nutrition are essential to the normal development of larvae until emergence as young adult bees. Larvae obtain all their nutrients from several glandular secretions provided by nurse bees during the first 5–6 days of the larval stage (Stell 2012). Afterward, larvae receive a mixture of jelly, honey, and pollen until day 9, when the worker cells are capped off and feeding stops (Crailsheim 1990). The developing larvae undergo pupation from day 13 until emergence at day 21 (Stell 2012). If, during development, bees have access to all essential nutrients but in limited quantities, their body size and weight will be reduced (Eishchen et al. 1982, Daly et al. 1995, Scofield and Mattila 2015). However, several studies have indicated that larvae can be successfully reared during periods of nutritional stress (Eishchen et al. 1982, Hoover et al. 2006, Li et al. 2014, Zheng et al. 2014, Moerman et al. 2015, Scofield and Mattila 2015). Inadequate larval nutrition during development results in a number of significant effects on adult life history traits such as reduced longevity (Eishchen et al. 1982, Li et al. 2014, Zheng et al. 2014, Scofield and Mattila 2015), impaired ovary development in workers (Hoover et al. 2006), impaired learning (Arien et al. 2015), increased susceptibility to pesticides (Mogren et al. 2019), impaired waggle dance communications, premature onset of foraging activities (Scofield and Mattila, 2015) and increased susceptibility of the larvae to the fungal parasite *Aspergillus flavus* (Foley et al. 2012). Therefore, larval nutrition impacts both larval development and adult performance.

Previously, larval nutrition was controlled at the colony level by experimental manipulation of either quantity (Eishchen et al. 1982, Hoover et al. 2006, Scofield and Mattila 2015) or quality (Kunert and Crailsheim 1988, Hoover et al. 2006, Manning 2006, Foley et al. 2012, Li et al. 2014, Zheng et al. 2014, Zhang and Xu 2015) of food sources available to nurse bees, or by artificially feeding larvae in vitro under controlled laboratory environments (Brodschneider et al. 2009, Zhang and Xu 2015). Under these scenarios, food sources that colonies had access to were either a mix of unknown pollen available from the environment, artificial feeds (protein supplements or liquid feeds), or a combination of both. In addition, most nutritional studies concentrated on the impact of protein content on larval development (Kunert and Crailsheim 1987, Hoover et al. 2006, Li et al. 2014, Zheng et al. 2014). Other studies investigated the impact of pollen components such as calcium (Zhang and Xu 2015), some minerals (Herbert and Shimanuki 1978), sugars (Kunert and Crailsheim 1987, Brodschneider et al. 2009), l-arginine (Herbert et al. 1970), and linoleic and oleic fatty acids (Manning 2006) on larval development. However, the effects of many other nutritional components, such as amino and fatty acids, and variations of minerals on larval development have not been fully investigated.

Pollen of different plant species varies greatly in their protein, fat (Roulston and Cane 2000, Somerville 2000, Manning 2001a, 2001b), and mineral content (Herbert and Miller-Hill 1987, Herbert 1992, Serra Bobvehi and Jorda 1997, Manning 2001b, Somerville and Nicol 2002). Such variability can influence the nutritional composition and quantity of the food produced by the nurse bees for the colony (Standifer 1967). For example, fat and carbohydrate content in royal jelly is affected to a greater extent by diet than is protein or water content (Wright et al. 2018). In newly emerged worker honey bees, seasonal changes to the larval diet may contribute to changes in the body composition of protein, triglycerides, fats, glucose, and glycogen levels (Kunert and Crailsheim 1988). However, the impact of specifically feeding different types of pollen on the development of larvae has never been investigated.

In this study, we hypothesized that nurse bees will effectively utilize the nutrients at the colony level from different pollen sources to support the optimal development of larvae until emergence. We selected 4 commercially important pollen sources, including 2 endemic species to Western Australia, jarrah (*Eucalyptus marginata* Sm.) and marri (*Corymbia calophylla* Lindl.), and 2 agricultural crops pollinated by honey bees, white clover (*Trifolium repens* L.) and canola (*Brassica napus* L.). These pollens vary markedly in nutritional content.

We predicted that nurse bees would regulate larvae nutritional needs at the colony level and be able to compensate for variations in nutrient composition in their diet to maximize the health of future generations of bees. We, therefore, expected minimal effects on the body composition of emerging bees.

## Materials and Methods

### Study Site

The study took place at 2 locations. At location 1, herein referred to as the “buildup phase” (near Oldbury 6121, Western Australia, Latitude: −32.2° S, Longitude: 115.9° E), experimental colonies were assembled and monitored for 6 weeks (Table 1). The colonies were then shifted to location 2, a commercial avocado orchard, herein referred to as the “pollination phase” (near Busselton, Western Australia, Latitude: −33.7° S, Longitude: 115.5° E), and monitored for a further 6 weeks.

**Table 1.** The experimental timeline is September–November 2017, and sample collection/measurements are indicated by “x”. Week 4 is when the first bees were born on the assigned feed sources

Phase	Sampling week	Bee weights	Body composition	Patty consumption
Buildup	1			
	2			
	3			
	4	x	x	x
	5			
	6	x	x	x
Pollination	7			
	8	x	x	x
	9			
	10	x	x	x
	11			
	12	x	x	x

### Colony Setup

Forty nucleus hives (herein referred to as colonies) were set up with 4 feed sources (marri, jarrah, clover, and canola) with 10 replicates each. All colonies were 4-framed, full-depth Langstroth colonies. Each contained 1 frame of stored pollen and 1 frame of stored honey (from 1 of the 4 assigned feed sources), a drawn frame, and a waxed foundation frame (no drawn wax). The pollen and honey frames were sourced from local beekeepers prior to the experiment during the peak flowering times of the source plants and stored at −20 °C. (Supplementary Table S1). All honey and pollen frames were weighed individually prior to the experiment. Approximately 800 ± 10 g of bees per colony were sourced from a commercial beekeeper (operating north of Geraldton, Western Australia). Six-month-old, open-mated sister queens (*Apis mellifera ligustica*) were purchased from a local queen breeder. In some colonies, queen failure occurred, and these were promptly replaced with same-aged queens of similar genetic stock. No treatments were applied to manage pests or diseases during the entire duration of the experiment. Each colony was fitted with an external ANEL pollen trap.

### Feed Sources

In addition to the stored honey and pollen frames, bees also had constant access to prepared pollen patties of the same plant species. Bulk pollen was purchased from beekeepers who had trapped pollen during nectar flows of jarrah (*Eucalyptus marginata* Sm.) represented by 41.0% [Note: Jarrah forests show evidence of hybrid species; therefore, pollen can be represented by closely related pollen grains that cannot confidently be assigned to jarrah (Kratz 2022)], marri (*Corymbia calophylla* Lindl.) represented by 63.2%, coastal pollen from an overwintering heathland site, later identified as being majority clover pollen (*Trifolium repens* L.) represented by 88.2%, and canola (*Brassica napus* L. Round-up Ready 43Y23) (Supplementary Table S1), represented by 89.8%. All pollen sources were stored at −20 °C prior to experiments. Honey was obtained from the same apiary sites as the additional pollen after commercial extraction, and 82.0%, 28.0%, 50.3%, and 41.1% of the pollen found in the honey respected marri, jarrah, clover, and canola, respectively (Kratz 2022).

Each pollen type was subsequently processed into patties (150 g each) by adding honey from the respective feed source in a ratio of 6:1 (pollen to honey by weight). Patties were stored at −20 °C, thawed prior to use in colonies, and fed weekly.

The patties were analyzed for nutritional composition at a NATA-accredited chemistry laboratory (ChemCentre, Resources and Chemistry Precinct, Bentley, WA, Australia) as follows.

#### Moisture

Moisture was determined by drying at 105 °C for 16 h as recommended by the American Association of Cereal Chemists (ADLM Method 44-15.02).

#### Ash

Ash is the total amount of minerals and silica content of feed materials. The ash content was determined by ashing the samples at 600 °C for 16 h (Thiex et al. 2012, AOAC method 942.05, AACC method 08-83). The ash is the weight of residue determined after cooling in a desiccator.

#### Minerals

The procedure of destroying the organic matter by digestion in acid for P, S, K, Na, Ca, Mg, Cu, Fe, Mn, Zn, and B in food samples is based on nitric/perchloric acid digestion to a temperature of 200–210 °C. Upon cooling, the mixture is diluted with deionized water, and the minerals are quantified using inductively coupled plasma atomic emission spectroscopy (ICP-AES) against primary standards (McQuaker et al. 1979). The method is certified according to the National Association of Testing Authorities, Australia (NATA).

#### Total Protein

Nitrogen is determined by placing a weighed subsample in a furnace at 850 °C and flushing it with oxygen for rapid combustion of the sample. Combustion products are collected. An aliquot taken from the combustion products is carried via a helium stream through various scrubber columns to remove carbon dioxide, oxygen, and vaporized water. The various forms of nitrogen are then converted to N_2_ by a catalyst and measured in a thermal conductivity cell. (The process is automated by the LECO Corporation) ChemCentre uses a LECO combustion analyzer for this purpose by AOAC using method 990.03 (AOAC 2000).

Nitrogen results are calculated and expressed as a percentage as received in the sample, which is then converted to protein equivalents using the protein factor 5.6 to obtain the percent of protein in the sample (Rabie et al. 1983). The final results are expressed as crude protein (CP).

#### Amino Acids

Total amino acids are determined after the first step of hydrolyzing the protein with 6 N HCl for 24 h to free the amino acids. After hydrolysis, the solution is diluted and filtered, and the HCl is neutralized with sodium hydroxide (Llames and Fontaine 1994). All amino acids are determined individually except cysteine and cystine, which are degraded to cysteic acid and reported as a combined outcome. Tryptophan is partially destroyed in 6 M HCl. Amino acids were determined by the method outlined by AOAC methods 994.12 (sulfur and regular) and 988.15 (tryptophan) (AOAC 1995). Since amino acids have low absorptivity in the UV/vis range, the analysis uses precolumn derivatization to form adjunct compounds which have high absorption in the UV and, therefore, can be determined by HPLC with a greater sensitivity.

#### Total Lipids

Pollen was freeze-dried, 3–5 g of sample were accurately weighed, and the ternary solvent mixture was added and high-speed blended with an IKA T25 Ultraturrex immersion blender. Total lipids, including phospholipids, were extracted using the ternary solution with a chloroform/methanol 2:1 ratio solvent extraction according to the method of Folch et al. (1957). Solvent ratios of chloroform, methanol, and water in the combined phases were maintained as close as possible to 8:4:3 (by volume) ratios to ensure appropriate lipid extraction based on selective polarity and then phase separation. The lower phase after separation was collected and made to a known volume, and an aliquot rotary evaporated at 37 °C to dryness and weighed. Gravimetric total lipid concentration was then extrapolated for the original sample.

#### Fatty Acids

Following the Folch extraction for total lipids, an aliquot of the phase-separated extract containing approximately 0.05 g of lipid was then saponified, methylated, and esterified using boron trifluoride as a catalyst. This was conducted under reflux conditions over a steam bath to create fatty acid methyl esters from the triacylglycerides, glycolipids, and phospholipids present in the oil (Christie 1989). Esterified samples were run on an Agilent 6890 Gas Chromatography with flame-ionization detection (GC-FID) with a dual column (ID 0.25 mm, film thickness 1.25 µm, Column 1 Film SGE BPX 70 length 30 m, Column 2 Film SGE BPX 90 length 30 m) setup with dean switch to selectively extend the resolution of fatty acid isomers measured. Retention times were compared to internationally certified reference standards, and after identification, peak areas were quantified (AOAC 1990). Individual fatty acid concentration is expressed as a percentage of the total fatty acids identified.

Key fatty acid groups such as total saturated and unsaturated, mono and polyunsaturated, trans, omega 3, and omega 6 classes of oils were determined based on the international definitions for each of these classes (FSANZ 2017).

### Emerging Bees

#### Collection

Only bees that were chewing through the cell capping and had, therefore, not consumed food other than during the larval stage were selected. Between 10 and 15 newly emerged bees were collected from each colony every second week. Collection began in week 4 when the first bees reared on the assigned feed sources emerged, and the experiment concluded in week 12 (Table 1). The bees were collected in 50 ml screw-top containers and kept on ice in the field. They were first freeze-killed and stored at −20 °C before being transferred to a laboratory freezer at −80 °C for further analyses.

#### Emerging bee weight

The bees were individually freeze-dried in Eppendorf tubes using a VirTis benchtop 2K freeze dryer until no further weight changes were observed. The bees were weighed inside the Eppendorf tube using a 0.001 g precision balance (Mettler Toledo, ML 104/01, Switzerland), and the dry weight was calculated by subtracting the Eppendorf tube weight from the total weight.

Immediately after freeze-drying and weighing, 50–60 individual bees in Eppendorf tubes were grouped by colony in postage envelopes. Batches of 4 envelopes were placed in a Ziploc bag containing 5 g of desiccant (Silica gel orange, SL421, chem-supply). The dried bee samples were then combined twice across 5 colonies per feed source (*n* = 2) for each week and transported to the ChemCentre (Building 500, Curtin University, Western Australia) for analysis.

#### Body composition

The dried bee samples were milled in a high-speed plunge mill. Approximately 1 g per sample was weighed and used for mineral, amino acid, total fat, and fatty acid analyses by ChemCentre, the same NATA-accredited chemical laboratory used for the feed sources.

### Patty Consumption

The dry weight of each of the patties (150 g), before feeding it to the colony, was obtained by weighing out subsamples of 5 patties (7–10 g) and drying these in an incubator at 60 °C. The moisture content was determined using a 0.001 g precision balance (AND, GX-400, Australia).

Each week, uneaten patties were collected and frozen in Ziploc bags at −20 °C following the same method of drying to determine dry weight consumption. The dry weight of patties consumed by individual colonies was then calculated on a daily basis (g/day), taking into account colony size. Colony size was determined by the number of capped brood cells per colony every second week from digital photographs and counted using the desktop software “Beestly” (available from www.cyency.com). Patty consumption (g/day/100 brood cells) was therefore analyzed every second week (Table 1).

### Data Visualization and Classification

The mineral composition of the patties was compared to estimated honey bee mineral requirements from pollen as described by Black (2006).

Amino acids were classified into essential and nonessential amino acids and compared to the minimal requirements of amino acids previously published by De Groot (1953). All amino acids are presented as a relative percentage of the total concentration of amino acids in each sample. The total protein content of emerged bees could not be determined due to technical difficulties. Hydroxyproline and taurine were excluded from the amino acid dataset of the bees because their values were too close to the detection limit of the GC–MS platform used for the analyses.

All unknown fatty acids were removed from the dataset, and only fatty acids that exceeded 0.1 g/100 g of the total fat content were reported in the results (Manning, 2006). All other known fatty acids are reported in Supplementary Tables S2 and S3.

Due to some technical difficulties in sample preparation, fewer minerals are reported for the bees than for the food sources. Potassium, sulfur, sodium, and iron are only reported in the food sources, whereas phosphorus, magnesium, calcium, zinc, copper, and manganese are reported in the food sources and the bees. In addition, boron was only analyzed in the bees. One replicate sample (bees reared on jarrah of the second sampling week) was an extreme outlier and excluded from the dataset.

Despite that the use of pollen traps restricted the access to foraged pollen from the environment, *Phase* (buildup or pollination) was considered as a factor because different types of nectars were collected by bees at each phase (Table 1) and therefore reflecting possible environmental influences on the results.

The bees collected in week 8 were fed as larvae during the “buildup phase,” however, they were born during the “pollination phase” because the colonies were shifted prior to their emergence. Therefore, data obtained for those bees were analyzed as part of the “buildup phase.” Sampling of emerging bees commenced at week 4, when the first hatching bees reared on the allocated feed source could be collected.

### Statistical Analysis

All statistical analyses were performed in R, version 3.5.2 (R Core Team 2020). All datasets were checked for normality and equal distribution of variance and analyzed by Linear Mixed Effects Models using the package “*nlme*” (Pinheiro et al. 2020). *Feed Source* and *Phase* were included as fixed factors in each model. *Colony* was included as a random factor for the analysis of bee weights and patty consumption. To account for the repeated sampling of bees from the same colonies, *Week* was nested within *Colony*. The data for patty consumption was normalized using a log transformation. One replicate colony of the jarrah feed source (for week 6 at the “buildup phase”) was removed as the capped brood count created an extreme outlier. However, removing the outlier did not change the overall outcome of the model. For the analysis of the body composition of emerging bees, *Week* was nested within the combined sample and was included as a random factor. One replicate sample for jarrah in week 4 was an extreme outlier and was removed for mineral analysis.

All data are presented as mean ± sem. A post hoc using Tukey’s pairwise comparisons was used to test for within-factor effects from the R package “*lsmeans*.” Results are presented as mean ± SE, and differences were deemed significant at *P* < 0.05.

## Results

### Composition or Proximal Analysis

#### Patties

The moisture content was approximately 30% in marri, clover, and canola patties and 25% in the jarrah patties. The ash content was the lowest (2.1%) in marri patties and highest in clover patties (2.8%) and intermediary for both jarrah and canola patties, 2.3% and 2.6%, respectively. Protein content ranged from 18% to 22% total protein (Table 2). The total fat content of the canola patties was 20.1%, which was more than twice that of the marri and jarrah patties and almost twice the fat content of the clover patties (Table 2).

**Table 2.** Summary of proximate analyses of marri, jarrah, clover, and canola patties and emerging bees feed on the food sources

Component	Patty				Bees			
	Marri	Jarrah	Clover	Canola	Marri	Jarrah	Clover	Canola
Moisture (%) ar^a^	31.3	24.6	29.1	31.4	83.9 ± 4.4	84.4 ± 3.8	84.1 ± 4.3	84.3 ± 3.9
Ash (%) db	2.1	2.3	2.8	2.6	4.4 ± 0.63	5.0 ± 0.50	4.3 ± 0.26	4.4 ± 0.60
Protein (%) db^b^^,^^c^	20.2	18.3	21.8	21.9	Unknown			
Fat (%) db	9.84	8.97	11.2	20.1	8.6 ± 0.46	9.0 ± 0.37	9.2 ± 0.31	9.3 ± 0.42

^a^ar, as received.

^b^db, on dry basis.

^c^Protein was calculated by multiplying %N × 5.6.

#### Bees

A total of 2199 emerging bees were collected across the 40 experimental colonies. Between 10 and 15 emerging bees were collected from each feed source across 7–10 colonies for every sampling week across the 2 phases (Table 2). Some colonies did not have any hatching bees when emerging bees were collected. The lack of emerging bees was due to queen failure in a colony (Table 3).

**Table 3.** The average number of hatching bees (mean ± SEM) collected from replicate colonies (number of colonies shown in brackets) from each of the 4 feed sources marri, jarrah, clover, and canola across sampling weeks within each location

Phase	Week	Marri	Jarrah	Clover	Canola
Buildup	4	10.7 ± 0.2 (10)	10.6 ± 0.2 (10)	10.5 ± 0.2 (10)	10.0 ± 0.0 (10)
	6	11.6 ± 0.4 (7)	12.8 ± 0.6 (9)	12.7 ± 0.7 (9)	12.0 ± 0.3 (10)
	8	11.9 ± 0.3 (8)	12.0 ± 0.2 (9)	11.6 ± 0.3 (9)	11.7 ± 0.4 (7)
Pollination	10	11.8 ± 0.4 (9)	12.7 ± 0.6 (9)	12.2 ± 0.3 (10)	12.8 ± 0.4 (10)
	12	12.8 ± 0.4 (10)	13.1 ± 0.4 (10)	11.7 ± 0.2 (9)	12.8 ± 0.4 (10)

The ash content of emerging bees ranged from 4.3% ± 0.26% in bees fed on clover to 5.0% ± 0.50% in bees fed on marri; however, there was no main effect of *Feed source* on ash content (*F* = 0.9, *df* = 3, *P* = 0.53). Similarly, the fat content of bees, which ranged between 8.6 ± 0.46% in bees fed on jarrah to 9.3 ± 0.42% in bees fed on canola, was not significantly different between feed sources (*F* = 0.8, *df* = 3, *P* = 0.54). *Phase* had a significant effect on ash content (*F* = 11.4, *df* = 1, *P* = 0.0022) and was significantly higher during the “buildup phase” (*P* = 0.0026), but no differences in fat content between phases were found (*F* = 0.9, *df* = 1, *P* = 0.33). There was no interaction between *Feed source* and *Phase* for the total ash (*F* = 1.6, *df* = 3, *P* = 0.21) and fat (*F* = 0.2, *df* = 3, *P* = 0.93) content for bees reared on marri, jarrah, clover, and canola feed sources.

### Minerals

#### Patties

Jarrah and clover patties were similar in potassium content and approximately 20% greater than for marri patties and ~ 30% greater than for canola patties (Table 4). Canola patties had the highest phosphorus, sulfur, calcium, and magnesium content. Clover patties were most similar in phosphorus content compared to canola, followed by jarrah and marri at ~35% lower. The sulfur content was very similar in marri, jarrah, and clover patties and only slightly greater in canola patties. Calcium content was particularly low in marri patties, about 50% less than in jarrah and clover patties and ~40% lower than in canola patties. Magnesium content was comparable between marri, jarrah, and clover patties but approximately 50% higher in canola patties. Sodium content was similar in marri, jarrah, and clover patties and ~25% lower in canola patties. Iron was ~25% lower in jarrah patties compared to the other patties. Manganese content was about twice as high in marri compared to clover and canola patties, and jarrah was ~35% greater than marri. Zinc content was similar between jarrah and clover but ~30% lower than marri and canola patties. Marri patties were highest in copper content (20 mg/kg), followed by jarrah patties at ~25% lower, clover patties at ~50% lower, and canola patties at ~60% lower.

**Table 4.** The mineral content of marri, jarrah, clover, and canola patties (dry weight) with reference to minimum requirements in pollen essential for the growth of bees (Black 2006) and the mineral content of emerging bees. Bold values are below the minimum levels in pollen (Black 2006)

Minerals (mg/kg)	Patties				Minimum requirements	Bees			
	Marri	Jarrah	Clover	Canola		Marri	Jarrah	Clover	Canola
Potassium	5,777	6,853	7,488	4,862	2,703	Unknown			
Phosphorus	3,414	3,613	5,293	5,782	2,872	6,516 ± 683	6,049 ± 300	6,286 ± 194	6,206 ± 532
Sulfur	2,626	1,994	2,195	3,285	2,027	Unknown			
Calcium	696	1,184	1,265	1,971	169	828 ± 80	778 ± 39	795 ± 25	738 ± 63
Magnesium	814	885	710	1,301	338	1,192 ± 115	1,105 ± 53	1,142 ± 28	1,103 ± 87
Sodium	**123**	**224**	**129**	**88**	338	Unknown			
Iron	131	86	105	122	51	Unknown			
Manganese	**41**	59	**16**	**26**	51	4 ± 0.4	3 ± 0.2	3 ± 0.3	3 ± 0.3
Zinc	59	44	46	62	34	91 ± 10	79 ± 4	88 ± 2	82 ± 6
Copper	20	15	9	8	8	23 ± 3	21 ± 1	23 ± 1	21 ± 2
Boron	—	—	—	—	—	26 ± 3	24 ± 3	27 ± 4	25 ± 3

#### Emerging bees

The concentration of minerals ranged from 3 to 6,516 mg/kg of dry body weight (Table 4). The most abundant minerals were phosphorus, magnesium, and calcium, representing between 98.3% and 98.4% of all measured minerals for emerging bees fed on the different feed sources. Manganese was the least abundant mineral, followed by copper, boron, and zinc (Table 4).

Copper content was the only mineral that increased significantly (*P* = 0.0046) from the “buildup phase” to the “pollination phase” (Table 4), with no main effect of *Feed source* (*F* = 0.9, *df* = 3, *P* = 0.52) on bees fed on marri, jarrah, clover, and canola patties. There was no interaction between *Feed source* and *Phase* except for phosphorus content (*F* = 4.4, *df* = 3, *P* = 0.012) and zinc (*F* = 3.6, *df* = 3, *P* = 0.26). These interactions were driven by one higher value in the dataset but when that value was excluded, the interaction was nonsignificant.

### Amino Acids

#### Patties

Individual amino acids made up between 0.7% and 21.5% of all measured amino acids in the patties.

##### Essential amino acids

**Fig. 1.**
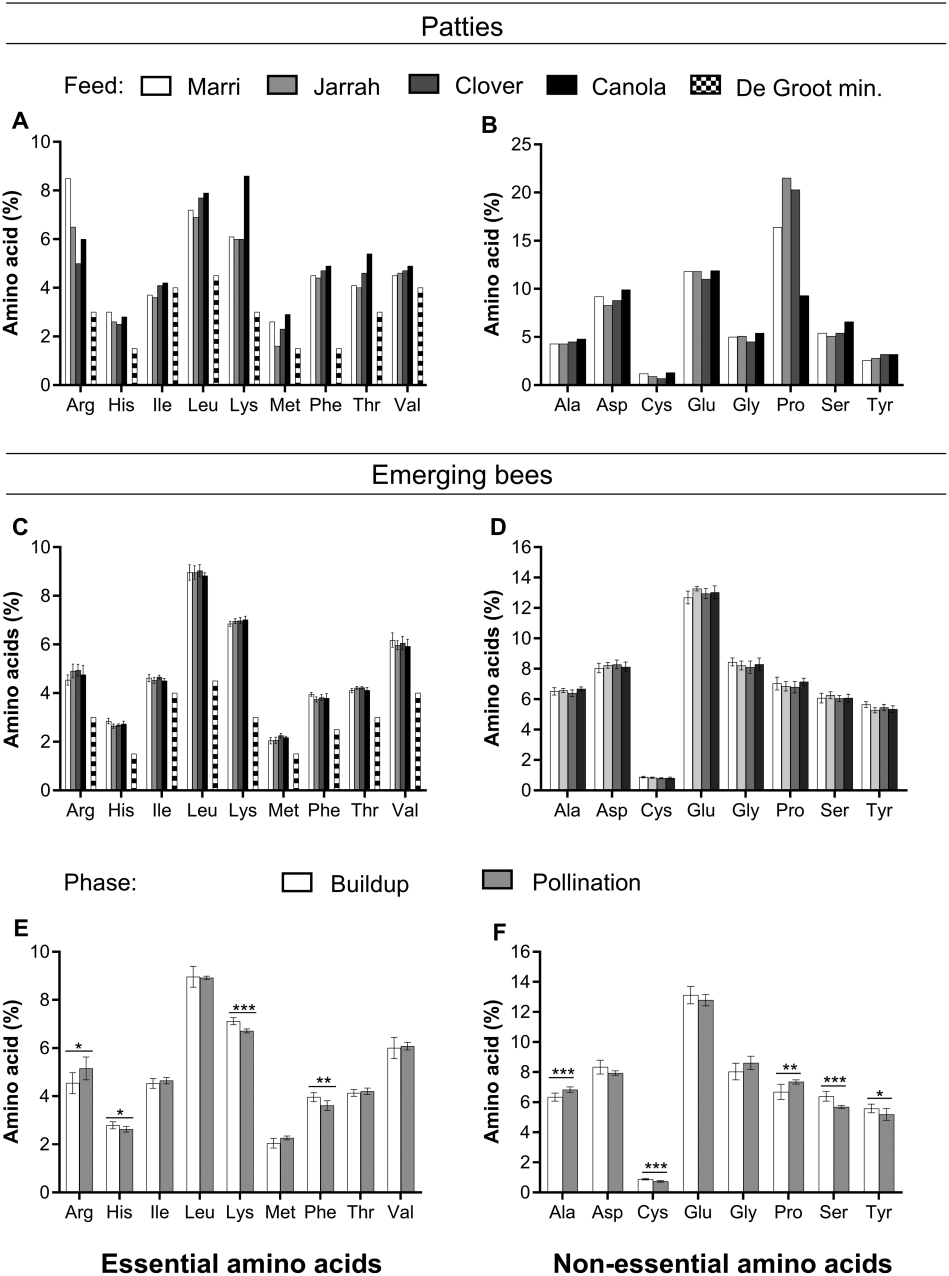
Amino acid profiles of patties and emerged bees (% ± SEM) fed on marri, jarrah, clover, and canola (light to dark bars) over 5 consecutive weeks. (Top) Essential amino acids A) and non-essential amino acids B) of patties. (Middle) Essential amino acids C) and non-essential amino acids D) of emerging bees. (Bottom) Essential amino acids E) and non-essential amino acids F) of emerging bees by location (the “buildup phase” in clear bars and the “pollination phase” in solid gray bars). Minimum levels of amino acids (striped bars) are defined by De Groot (1953). Arg, arginine; His, histidine; Ile, isoleucine; Leu, leucine; Met, methionine; Phe, phenylalanine; Thr, threonine; Val, valine; Ala, alanine; Asp, aspartic acid; Cys, cysteine/cysteine; Glu, glutamic acid; Gly, glycine; Pro, proline; Ser, serine; Tyr, tyrosine. An asterisk indicates significant differences (*P < 0.05, **P < 0.01, ***P < 0.001) from Tukey’s Comparison of Means.

Essential amino acids (Fig. 1) made up 44.2%, 40.2%, 41.6%, and 47.6% of the marri, jarrah, clover, and canola patties, respectively (calculated from the total weight of the patty). Within the essential amino acids, arginine, lysine, methionine, and threonine showed a 41%, 30%, 45%, and 26% difference between the highest and lowest measurements across feeds (Fig. 1). All other essential amino acids varied by 10%–17%.

##### Non-essential amino acids

Nonessential amino acids (Fig. 1) represented 55.9%, 59.8%, 58.4%, and 52.4% of the marri, jarrah, clover, and canola patties receptively (calculated from the total weight of the patty). Within the nonessential amino acids, cysteine/cystine, proline, and serine showed a 31%, 57%, and 23% difference between the highest and lowest measurements across feeds. All other nonessential amino acids varied by 8%–19% (Fig. 1).

#### Bees

The amount of individual amino acids measured in emerging bees varied between 0.80% for cysteine/cystine and 13.3% for glutamic acid of all measured amino acids.

##### Essential amino acids

Essential amino acids of bees represented, on average, 43.8%–44.7% across feed sources (Fig. 1).

##### Non-essential amino acids

Non-essential amino acids of bees represented, on average, 54.8%–55.5% (Fig. 1).

There was no interaction between *Feed source* and *Phase* for any of the amino acids. There was an effect of *Phase* on amino acids: arginine (*F* = 6.5, df = 1, *P* = 0.016), histidine (*F* = 5.3, df = 1, *P* = 0.029), lysine (*F* = 34.1, df = 1, *P* < 0.0001), methionine (*F* = 6.0, *df* = 1, *P* = 0.021), phenylalanine (*F* = 11.8, df = 1, *P* = 0.0019), alanine (*F* = 14.1, df = 1, *P* = 0.001), cysteine (*F* = 24.8, df = 1, *P* < 0.0001), proline (*F* = 0.0084, df = 1, *P* = 0.0084), serine (*F* = 21.5, df = 1, *P* = 0.0001), and tyrosine (*F* = 5.4, df = 1, *P* = 0.027) but not on any other amino acids (Fig. 1). Amino acids that were detected in increasing amounts from the “buildup phase” to the “pollination phase” included arginine (*P* = 0.015), methionine (*P* = 0.020), alanine (*P* < 0.001), and proline (*P* = 0.007). Amino acids that were detected in decreasing amounts from the “buildup phase” to the “pollination phase” included histidine (*P* = 0.030), lysine (*P* < 0.0001), phenylalanine (*P* < 0.005), cysteine (*P* < 0.001), serine (*P* < 0.001), and tyrosine (*P* = 0.024).

### Fatty Acids

#### Patties

**Fig. 2.**
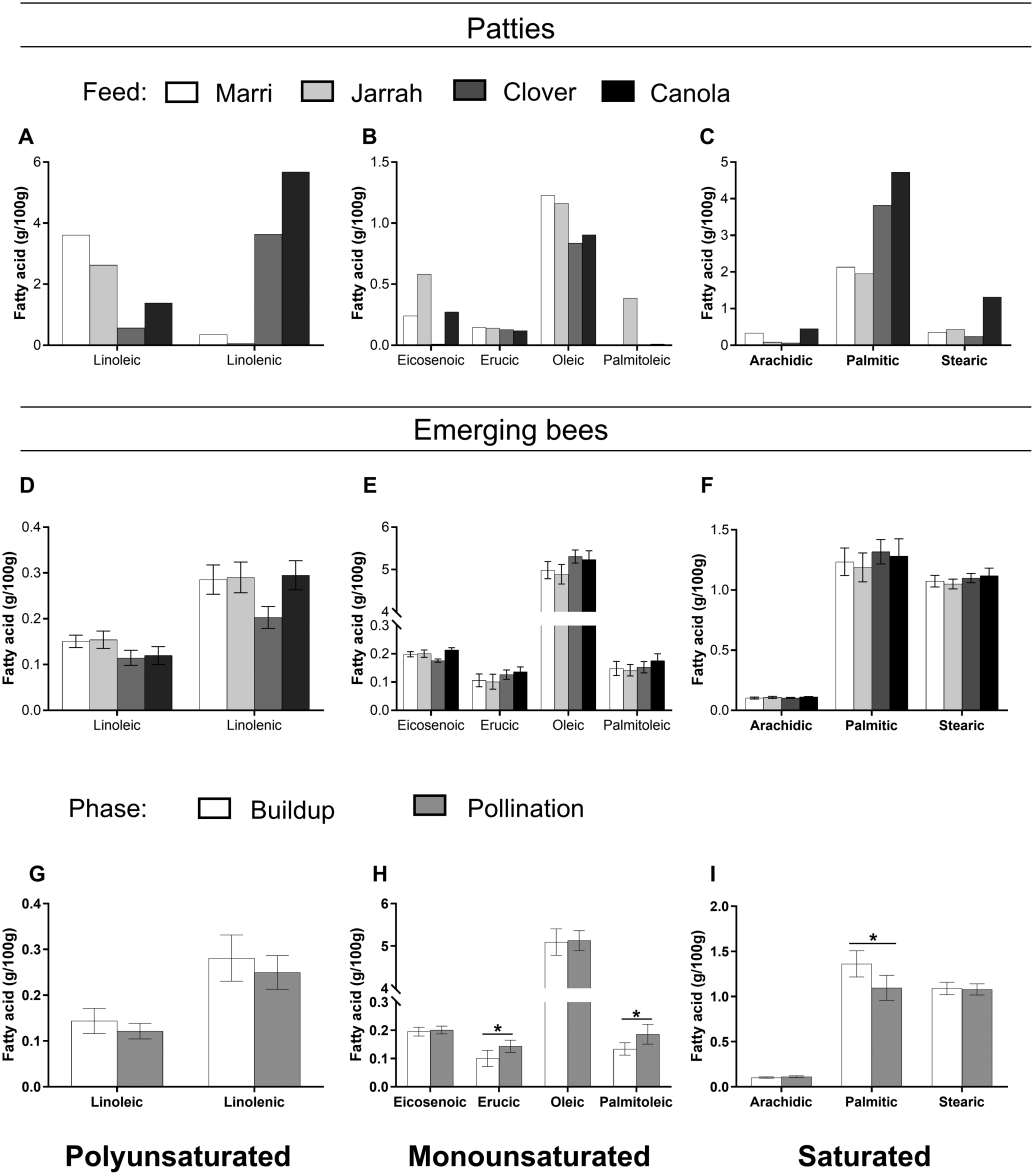
Fatty acid profiles of patties and emerged bees (g/100 g ± SEM) fed on marri, jarrah, clover, and canola (light to dark bars) patties over 5 consecutive weeks. A) Polyunsaturated fatty acids, B) monounsaturated fatty acids, and C) saturated fatty acids of patties, D) polyunsaturated fatty acids, E) monounsaturated fatty acids, and F) saturated fatty acids of emerging bees by feed source. G) Polyunsaturated fatty acids, H) monounsaturated fatty acids, and I) saturated fatty acids of emerging bees by location (the “buildup phase” in clear bars and the “pollination phase” in solid gray bars). An asterisk indicates significant differences (*P < 0.05, **P < 0.01, ***P < 0.001) from Tukey’s Comparison of Means.

All patty types varied in the number of fatty acids that made up most of the total fat content above 0.1 g/100 g, a cutoff point as per Manning (2006). In marri, jarrah, clover, and canola patties, 11, 9, 8, and 17 fatty acids, respectively, represented 84.5%–93.1% of the fat content. Jarrah patties were low in linolenic acid (0.05%) compared to the other patty types and were 85% lower than the marri patty. Both marri and canola patties had the highest arachidic acid content, and eicosenoic acid content was lowest in clover. Palmitoleic acid was highest in jarrah patties, and heneicosanoic acid was highest in clover patties. Fatty acids that exceeded 0.1 g/100 g in both the patties and the emerging bees are presented in Fig. 2.

##### Essential fatty acids

The concentration of linoleic acid was the greatest in marri patties and was similar to that in jarrah patties. The concentration of linoleic acid in marri patties was about twice that in canola patties and about 4 times as high when compared to clover patties (Fig. 2). The concentration of linolenic acid was the greatest in canola patties, at 5.68 g/100 g, followed by that in clover patties (~2/3rd the amount). Minimal concentrations of linolenic acid were detected in marri and jarrah patties (<0.5 g/100 g, Fig. 2).

##### Non-essential fatty acids

All 4 monounsaturated nonessential fatty acids, eicosenoic, erucic, oleic, and palmitoleic (above 0.1 g/100 g), were detected in the jarrah patties (Fig. 2). Palmitoleic acid was below the detection limit of 0.05% in marri, clover, or canola patties (Supplementary Table S2).

Amongst the saturated nonessential fatty acids detected, palmitic acid was the most abundant, followed by stearic and arachidic fatty acids (Fig. 2). The concentrations of palmitic acid in clover patties were about 25% less than measured in canola patties. The concentrations of stearic and arachidic acids were at least double in canola patties than in the other patties (Fig. 2).

#### Bees

Nine of the fatty acids in emerging bees represented 3 groups: polyunsaturated fatty acids (linoleic and linolenic), monounsaturated fatty acids (eicosenoic, erucic, oleic, and palmitoleic), and saturated fatty acids (arachidic, palmitic, and stearic acids). They represented 90.2%–93.2% of all fatty acids with a concentration greater than 0.1 g/100 g. No significant interaction between *Feed source* and *Phase* was found in any fatty acids. *Phase*, however, had a significant effect on the monounsaturated fatty acids erucic (*F* = 10.4, df = 1, *P* < 0.005) and palmitoleic (*F* = 14.1, df = 1, *P* < 0.001) and on the saturated arachidic (*F* = 4.3, df = 1, *P* = 0.048) and palmitic acid (*F* = 11.5, df = 1, *P* = 0.002). Fatty acids that were detected in increasing amounts from the “buildup phase” to the “pollination phase” included erucic (*P* < 0.005), palmitoleic (*P* < 0.001), and arachidic (*P* = 0.043). The only fatty acid that was detected in decreasing amounts from the “buildup phase” to the “pollination phase” was palmitic (*P* < 0.005). There was no effect of *Feed source* on any of the fatty acids (Fig. 2).

Nineteen other fatty acids represented the remaining 6.8%–9.8% of all fatty acids, of which 10 fatty acids were below the detection limit for at least one of the feed sources (Supplementary Table S2).

### Patty Consumption

There was a main effect of *Feed source* (*F* = 38.9, df = 1, *P* = 0.018) on patty consumption, with colonies feeding on clover patties consuming significantly less feed compared to colonies fed on marri patties (*P* = 0.043), just not significantly different to jarrah patties (*P* = 0.05) and not to canola patties (*P* = 0.83).

**Fig. 3.**
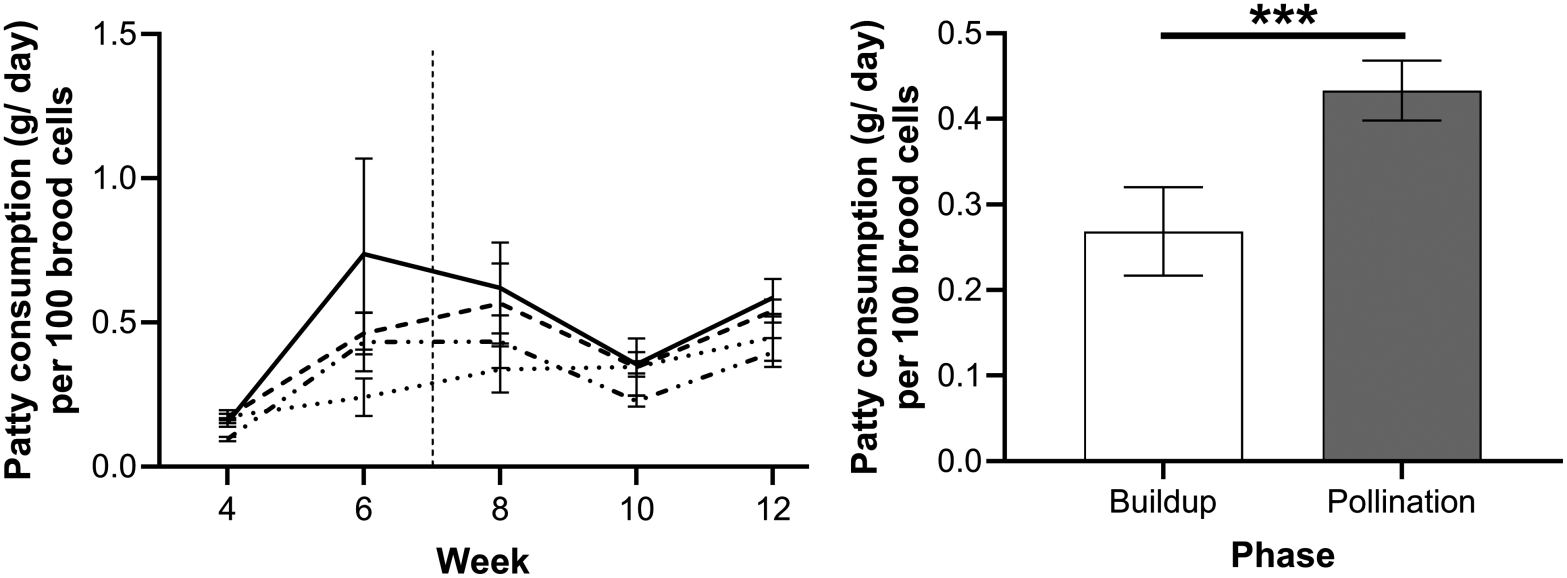
Average patty consumption per 100 brood cells accounting for colony size (g/day ± SEM) of emerged bees fed on marri (dashed line), jarrah (solid line), clover (dotted line), and canola (dashed and dotted) (10 colonies/feed source). (Left Panel) The horizontal dashed line indicates the change in location from the “buildup phase” (left) to the “pollination phase” (right). (Right Panel) Average patty consumption across locations. An asterisk indicates significant differences (*P < 0.05, **P < 0.01, ***P < 0.001) from Tukey’s Comparison of Means.

Patty consumption significantly increased across the phases (*F* = 38.9, df = 1, *P* < 0.001) (Fig. 3). It must be noted that for the jarrah feed source in week 6, one colony had a lower brood count and, therefore, raised the mean of the overall patty consumption with a corresponding larger standard error. The mean for bees feeding on jarrah patties throughout the experiment was 0.42 g/100 g ± 0.11 of patty per 100 brood cells. No interaction between *Feed source* and *Phase* was found for patty consumption (*F* = 0.12, df = 3, *P* = 0.95).

### Emerging Bee Weights

There was no effect of *Feed Source* (*F* = 1.1, *df* = 3, *P* = 0.35) on the dry weights of bees, but there was an overall effect of *Phase* (*F* = 10.6, df = 1, *P* = 0.0014) (Fig. 1). Bee weights were significantly lower (*P* < 0.001) during the “buildup phase” (dry weight: 17.7 ± 0.16 mg) than during the “pollination phase” (dry weight: 18.4 ± 0.08 mg). There was no significant interaction between *Feed Source* and *Phase* (*F* = 0.3, df = 3, *P* = 0.82).

## Discussion

Nurse bees of colonies provided with 4 commercially important pollen sources from the forest species, marri and jarrah, as well as 2 agricultural species, white clover and canola, were able to support the development of bees until emergence. The differences in the food composition of patties had no influence on the body weight of bees, their mineral, amino acid, or their fatty acid composition.

### Feed Source

Feed sources were most similar in their total protein content but differed in one amino acid, isoleucine, that was below minimum requirements in the jarrah and marri patties, according to De Groot (1953). The biggest differences between the feeds were generally seen in mineral content, total fat content, and the quantity of individual fatty acids. The 2 forest species marri and jarrah were more similar in their nutritional makeup compared to the agricultural species of clover and canola.

The CP content of the 4 feed sources reported here was slightly lower than those previously published. The ratio of pollen to honey that was used to make the patties explains about 14% of that difference. The CP content of patties made of marri pollen (*Corymbia calophylla* Lindl.) was 20% compared to ~27% reported for marri pollen (Bell et al. 1983, Manning 2001b). Similarly, ~20% CP has been reported for jarrah pollen (*Eucalyptus marginata* Sm.) (Bell et al. 1983, Manning 2001b), and our samples measured 18% CP. The patties made with clover pollen (*Trifolium repens* L.) contained 22% CP, and 26% CP has been published for pollen of clover (Crailsheim et al. 1992, Manning 1995, Somerville 2001). The patties made with canola pollen (*Brassica napus* L.) contained ~22% CP compared to 23%–27% published for canola pollen (Bell et al. 1983, Manning 2001b). The unknown amount of nectar that honey bees add to pollen during foraging could also explain some of the differences observed in the CP from our data compared to previously published data (Wright et al. 2018), along with the variation in purity of a pollen source even during a major flowering event of a single species. The honey’s contribution to the total CP of the patties was negligible as honey contains approximately 0.5% protein (Bogdanov et al. 2008).

Isoleucine was low in both marri and jarrah patties. In fact, low levels of isoleucine have been reported in pollen of jarrah and many eucalypt species (Rayner and Langridge, 1985, Somerville, 2000, Manning 2001b, Somerville and Nicol 2006). Generally speaking, though, marri pollen has been identified as nutritionally complete in amino acids for honey bees (Manning 2001b). Differences may again be explained by the floral diversity and by the way the patties were prepared, creating a “dilution” of nutrients per gram of pollen after being mixed with honey.

The total ash content, a proxy for total mineral content, was the highest in patties made from agricultural pollen types, being higher in clover than in canola. Patties made from the pollen of jarrah and marri trees were lower in ash content. The difference in the total mineral content and the amount of individual minerals of the 4 types of patties may be explained by the annual addition and use of mineral fertilizers on agricultural farmland compared to natural forest soils. Jarrah trees grow in soils derived from laterite, which is also similar for marri. However, marri can also be found growing in deeper sandy soils overlying granite (Smith 1969).

Jarrah and marri patties showed similar mineral profiles except for calcium being about twice as high in jarrah patties, which has been reported previously (Manning 2001b). In all patties, mineral content was well above the estimated minimum requirements for bees, except for sodium and manganese in marri, clover, and canola patties (Black 2006). However, the minimum requirements for sodium might have been overestimated in honey bees because, as in mammals, sodium accumulates in the body tissues of bees (Black 2006).

Total fat content differed between the patties of different floral origins. Canola and white clover patties contained higher total fat content compared to marri and jarrah patties. All the values for fat content reported were mostly higher than those previously reported for pollen. The fat content of canola (*Brassica napus* L.) patties measured in the present study (20.1%) was in the middle range compared to previously reported values of 7.3%–31.7% (Evans et al. 1987, Somerville 2001, Odoux et al. 2012). The fat content for clover patties (11.2%) was higher than that reported previously for pollen (2.7% and 3.8% in *Trifolium repens* L. white clover: Roulston and Cane 2000, 2.5% for *Trifolium* species: Somerville 2001). Fat content in pollen collected by bees from marri, jarrah, and eucalypt species, in general, has been reported as less than 2% (Somerville, 2000, Manning 2001a). The greater fat content for both jarrah (9%) and marri (9.8%) patties in this study, as well as for clover patties and to some degree also for canola patties, as mentioned above, is likely a result of the lipid extraction method used. The extraction method, a total lipid extraction that includes triacyl glyceride, neutral and phosphor lipids, and whether or not the pollen grain wall was fractured prior to lipid extraction are all possible contributing factors when comparing the fat content of various pollen types between studies (Evans et al. 1987, Scott and Strohl 1962). It has to be noted that the extraction efficiency, however, does not affect the fatty acid composition (Iverson et al. 2001). The level of some individual fatty acids in the patties varied between feed sources. However, the fatty acid requirements are still not well understood. Two essential fatty acids, linolenic and linoleic, are known to impact the health of bees (Hulbert and Abbott 2011, Arien et al. 2015) and were different between the 4 feed sources.

### Emerging Bees

While the nutrient content of the patties presented some differences in the nutritional composition between the floral sources of pollen, the body composition of the emerging bees fed on the different feed sources did not differ in essential amino acids, total mineral content, fat and fatty acid content, but differed marginally in feed consumption. An effect of *Phase* (“buildup” phase vs. “pollination phase”) was observed for body weight, copper, several amino acids, and fatty acids, independently of the type of patties available.

Nurse bees regulated the nutrients fed to larvae independent of the food source. Adult bees are known to be able to vary their food intake of macronutrients in order to meet an optimal balance of nutrients, such as protein and fat (Stabler et al. 2021); this appears to be the case with nurse bees. Alternatively, nurse bees, to some degree, sacrifice their own body stores of protein for the survival of the next generation (De Groot 1953, Kleinschmidt and Kondos 1976). Such feeding strategies can help to explain how honey bees can regulate the nutrients of larval food in an environment that either provides pollen resources of varying nutritional quality to choose from or reduced levels of some nutrients. In the current study, the most likely mechanism that allowed bees to compensate for lower levels of isoleucine in jarrah and marri feeds was increased consumption of the feed, even though this was not statistically significant. This may also be true for the total lipid content and that of individual fatty acids that bees seemed to regulate for all feeds. However, the requirements of specific fatty acids for honey bees are still understudied, and the lipids in this study may have been sufficient to meet the nutritional requirements of the bees. For example, artificially increasing linoleic and oleic acid content in marri pollen did not increase the longevity of bees in cage experiments (Manning 2006). Future studies could consider comparing the body composition of emerging bees along with those of nurse bees and any flow-on effects on later adult life and successive colony lifecycles.

There was also no difference in the mineral composition between the emerged bees reared on the 4 types of patties in this study. The lack of difference was not expected, especially for calcium, as a previous study has suggested that the mineral content of pollen could influence the mineral composition of emerging bees (Manning 2002). Without established mineral requirements available for bees, we can only speculate that the requirements have been met for all minerals by all 4 food sources on offer, even for manganese, which was low in the patties according to published mineral estimates (Black 2006). The mineral composition of emerged bees in the present study was similar to that previously reported for copper, magnesium, and zinc but were higher by ~40%, ~80%, and ~25% for calcium, boron, and manganese, respectively (Black 2006, calculated from Manning 2002) and lower by ~30% in phosphorus (Manning 2002).

Interestingly, bees consumed less clover patty than other patties from other floral sources, possibly because of differences in the attractiveness and digestibility of the pollen. While there is no data on the digestibility of each of the food sources, pollen digestibility can vary between pollen types depending on pollen morphology, pollen size, and, more specifically, the pollen wall structure and the mechanisms by which the nutrients are released from the grain’s protoplasm (Stanley and Linskens 1965, Kroon et al. 1974, Peng et al. 1986, Roulston et al. 2000). If clover pollen is more digestible, then bees would have needed to consume less to meet their dietary requirements. It is unlikely that tannins present in jarrah or marri patties were responsible for low digestibility, causing increased consumption of the feeds, because tannins are known to rather have a toxic than inhibiting effect on nutrient uptake in insects (Barbehenn and Peter Constabel 2011, Salminen and Karonen 2011, Ibanez et al. 2012). Investigations on the digestibility of marri, jarrah, clover, and canola pollen would help to understand the differences in patty consumption between the feed sources. Future studies should also consider the influence of other secondary compounds, which in bees can affect the attractiveness of certain nectars (Afik et al. 2006, Wright et al. 2013) and food intake in other insects (Singer et al. 2009).

The increase in patty consumption from one *Phase* to the next could be due to stored food sources becoming exhausted over time, requiring colonies to consume a larger amount of the patties, and also because brood area will generally expand if food sources are abundant. However, the similarity in body composition of emerged bees strongly suggests that nurse bees were able to digest and regulate the nutrients required for feeding the larvae from all feed sources.

An effect of *Phase* was observed for body weight, copper, several amino acids, and fatty acids, independently of the type of patties available. Emerged bees at the “buildup phase” were smaller compared to emerged bees at the “Pollination phase.” Body weight and size are affected by nutrient availability (Kunert and Crailsheim 1988, Brodschneider and Crailsheim 2010, Scofield and Mattila 2015). As there was no effect of the feed sources on body size, other factors could have influenced the nutritional status of developing bees between the 2 phases of the experiment. One such factor could be the number of nurse bees to larval ratio, which influences the time nurse bees spend feeding larvae. In general, larger colonies have been found to rear heavier workers (Levin and Haydak 1951). Worker bees reared at nurse bee to larvae ratios of 0.5:1, 1:1, and 5:1 are smaller than workers reared at ratios of 100:1 (Daly et al. 1995). Worker bee weight and longevity increase with greater nutritional investment of nurse bees per larva (Eishchen et al. 1982). At the “buildup phase,” the colonies were still established after having been assembled after being shaken from bee packages and the addition of young queens at week 1. Most of the bees provided at the start of the experiment were nurse bees. However, the division of labor between nestmates of similar age occurs during the first 4–13 days after the establishment of a new colony (Manning 2006). Consequently, the number of nurse bees tending to eggs that the queen had already started laying was reduced. At the “pollination phase,” the colonies were already established and had increased in population size, and therefore, they were most likely to have a greater nurse bee to larval ratio. The larvae reared under these conditions were likely to be better fed and cared for, allowing bees to either grow bigger in size or accumulate more nutritional stores.

The change in body weight between the 2 *Phases*, over the whole duration of the experiment, did not influence the micronutrient composition of the bees as expressed per unit of body mass. However, emerging bees at the “buildup phase” had a greater ash content than emerging bees at the “pollination phase,” and copper was significantly greater in emerging bees at the “pollination phase.” Throughout the experiment, the colonies had free access to nectar. At the “pollination phase,” in addition to nectar from several common weed species, colonies had access to nectar from a 222-ha avocado crop that was in full bloom during the experiment. The higher copper levels in bees collected from the “pollination phase” could be explained by the availability of avocado nectar. Avocado nectar, and thus honey derived from it, is rich in a wide range of minerals, including potassium, phosphorus, magnesium, sulfur, iron, and copper, compared to some other honey (Afik et al. 2006). The higher mineral content available from avocado nectar could have contributed to the increased body copper content across all feed sources seen at the “pollination phase.” Other minerals present in avocado nectar and honey did not increase between the “buildup phase” and the “pollination phase,” possibly because of differences in assimilation between minerals. Why the overall ash content was significantly greater at the “pollination phase” is not known, and it may reflect an increased biogenic silica content and, hence, a difference in exoskeleton strength and composition. More research is needed to study how such differences in mineral nutrition could affect bee health and colony performance.

There was an effect of *Phase* on the concentration of several amino and fatty acids of emerged bees. However, the effect was not consistent across all amino acids. Some amino acids increased, and some decreased from the “buildup phase” to the “pollination phase.” These complex variations in body composition could be the result of differences in the availability of nutrients due to changes in the production of worker jelly produced by the nurse bees. Seasonal variations in weight and protein content of emerging bees have been linked to the availability of food sources from the environment (Kunert and Crailsheim 1988). Royal jelly, the larval food specifically for queen bees, has been found to vary largely in total fat and carbohydrate content between studies done in different countries, while total protein was found to be stable (Wright et al. 2018). It would be interesting to investigate how much queen and worker jelly composition varies in its individual amino and fatty acid composition and the possible effects on emerging bees with the same or similar feed sources.

Some differences in the amino and fatty acid composition of emerged bees may be explained by the fact that pollen traps do not remove all pollen collected by foragers. In this study, bees were readily trapped in their collected pollen in the traps, but bees were occasionally observed to cleverly adjust their leg position and carry pollen intact on their legs through the perforated barrier inside the traps. However, there was no evidence that the bees were storing the pollen in abundance. Nevertheless, small quantities of environmental pollen may have contributed to some variation in the amino and fatty acid composition of emerged bees in this study.

Overall, nurse bees were shown to successfully raise young adult workers from the larval stage until emergence, when artificially fed on various natural feed sources that differed in their nutritional composition, even under pollen-restricting conditions. Our results suggest that the availability of pollen may be more important than its composition as long as bees have access to all essential nutrients.

## Supplementary Material

toae045_suppl_Supplementary_Tables_S1-S3
