# Supplementary material for: Nurse bees regulate the larval nutrition of developing workers (Apis mellifera) when feeding on various pollen types
Source: J Econ Entomol. 2024 Apr 12;117(3):683–95. doi: 10.1093/jee/toae045 (PMC11163459; doi:10.1093/jee/toae045)
Supplement: toae045_suppl_Supplementary_Tables_S1-S3 [file toae045_suppl_supplementary_tables_s1-s3.docx]

**SUPPLEMENTARY MATERIAL**

**Table S1.** Details on the origin of the feeds (frames, bee-collected pollen and honey).

| **Feed type** | **Feed source** | **Sampling date** | **Chemicals used** |  | **Genetically modified** | **Location** | **Apiary site** |
| --- | --- | --- | --- | --- | --- | --- | --- |
| Frames (pollen and honey) | Marri | 6th Mar 2017 | no |  | - | Jarrahdale | AS 4594 |
| Bee-collected pollen | Marri | Feb-Mar 2017 | no |  | - | Jarrahdale | AS 4594 |
| Honey | Marri | Feb-Mar 2017 | no |  | - | Jarrahdale | AS 4594 |
| Frames (pollen and honey) | Jarrah | 16th Dec. 2016 | no |  | - | Jarrahdale | AS 3895 |
| Bee-collected pollen | Jarrah | 6th Jan 2017 | no |  | - | Jarrahdale | AS 4594 |
| Honey | Jarrah | 6th Jan 2017 | no |  | - | Jarrahdale | AS 4594 |
| Frames (pollen and honey) | Clover | early Aug 2017 | no |  | - | North of Jurien Rd, Jurien Bay | Private site |
| Bee-collected pollen | Clover | Sep-16 | no |  | - | North of Jurien Rd, Jurien Bay | Private site |
| Honey | Clover | Sep-16 | no |  | - | North of Jurien Rd, Jurien Bay | Private site |
| Frames (pollen and honey) | Canola | 8/11/2017 | yes |  | Round- up Ready 43Y23 | East Chapman Rd, Chapman Valley | Private site |
| Bee-collected pollen | Canola | 11th Aug 17 | yes |  | Round- up Ready 43Y23 | East Chapman Rd, Chapman Valley | Private site |
| Honey | Canola | 11th Aug 17 | yes |  | Round- up Ready 43Y24 | East Chapman Rd, Chapman Valley | Private site |

**Table S2.** Quantity of fatty acids of marri, jarrah, clover and canola patties. Fields highlighted in grey represent values greater than 0.1 g/100 g of dry material. ND: not detectable = values below the detection limit of 0.05%.

| **Fatty acids** | **Marri** | **Jarrah** | **Clover** | **Canola** |
| --- | --- | --- | --- | --- |
| Linoleic | 3.61 | 2.63 | 0.57 | 1.39 |
| Palmitic | 2.13 | 1.95 | 3.82 | 4.73 |
| Oleic | 1.23 | 1.16 | 0.84 | 0.9 |
| Linolenic | 0.36 | 0.05 | 3.64 | 5.68 |
| Stearic | 0.35 | 0.44 | 0.24 | 1.31 |
| Arachidic | 0.34 | 0.08 | 0.07 | 0.45 |
| Capric | 0.25 | 0.19 | ND | ND |
| Eicosenoic | 0.24 | 0.58 | 0.01 | 0.27 |
| Nervonic | 0.2 | 0.08 | 0.12 | 0.32 |
| Erucic | 0.15 | 0.14 | 0.13 | 0.12 |
| Myristoleic | 0.1 | 0.1 | 0.06 | 0.02 |
| Heptadecenoic | 0.06 | 0.09 | 0.01 | 0.02 |
| Cerotic | 0.05 | 0.04 | 0.04 | 0.13 |
| Octacosanoic | 0.05 | 0.02 | ND | 0.1 |
| Triacontanoic | 0.05 | 0.01 | 0.02 | 0.1 |
| Lignoceric | 0.04 | 0.02 | 0.03 | 0.09 |
| Arachidonic | 0.02 | 0.03 | ND | 1.01 |
| Eicosatrienoic | 0.02 | 0.04 | 0.03 | 0.06 |
| Tric.Doc | 0.02 | 0.02 | 0.03 | 0.04 |
| Eicosadienoic | 0.02 | 0.05 | ND | ND |
| Lauric | 0.02 | 0.03 | ND | ND |
| Pentadecenoic | 0.02 | 0.03 | 0.02 | 0.03 |
| Docosahexaenoic | 0.01 | 0.02 | ND | 0.01 |
| Docosapentaenoic | 0.01 | 0.02 | 0.01 | 0.1 |
| Palmitoleic | ND | 0.39 | ND | ND |
| Heptacosanoic | ND | 0.01 | ND | ND |
| Eicosapentaenoic | ND | ND | 0.03 | ND |
| Docosadienoic | ND | ND | 0.01 | ND |
| Heneicosanoic | ND | 0.04 | 0.63 | 0.04 |
| Behenic | ND | ND | 0.08 | 0.15 |
| Petroselinic | ND | ND | 0.06 | 0.68 |
| Myristic | ND | 0.01 | ND | 1.27 |

**Table S3.** Minor fatty acids (mg/100 g) of emerged bees reared on marri, jarrah, clover and canola feed sources. ND: not detectable = values below the detection limit of 0.05%.

| **Fatty acid (mg/100g)** | **Marri** | **Jarrah** | **Clover** | **Canola** |
| --- | --- | --- | --- | --- |
| Elaidic/Petroselinic | 21.5 ± 24.2 | ND | ND | ND |
| Cerotic | 30.4 ±1.3 | 29.5 ± 1.6 | 31.7 ± 1.7 | 32.7 ± 2.1 |
| Docosadienoic | 47.1 ± 21.6 | 51.8 ± 16.9 | 29.3 ± 13.1 | 32.6 ± 9.8 |
| Octacosanoic | 15.3 ± 1.1 | 15.7 ± 1.1 | 15 ± 1.2 | 16.4 ± 1.2 |
| Docosapentaenoic | 11.8 ± 3.0 | 10.9 ± 2.8 | 10.6 ± 3.1 | 9.2 ± 2.8 |
| Arachidonic | 7.1 ± 1.6 | 5.2 ± 1.2 | ND | 5.9 ± 1.3 |
| Behenic | 6.8 ± 3.2 | 6.5 ± 3.0 | 7.3 ± 3.8 | ND |
| Capric | ND | ND | 4.6 ± 0.2 | 5.7 ± 1.4 |
| Docosahexaenoic | 5.3 ± 1.1 | 5.2 ± 1.2 | ND | 5.3 ± 1.0 |
| Eicosadienoic | 6.3 ± 1.3 | 4.9 ± 0.7 | 6.0 ± 1.4 | 6.8 ± 1.7 |
| Eicosatrienoic | ND | 6 ± 1.2 | 6.1 ± 1.0 | 6.2 ± 1.0 |
| Heneicosanoic | 5.9 ± 1.3 | 5.8 ± 1.5 | ND | ND |
| Heptacosanoic | 7.3 ± 2.1 | 5.4 ± 1.0 | ND | ND |
| Heptadecenoic | 6.6 ± 1.5 | 6.5 ± 1.5 | 5.1 ± 0.8 | 7.3 ± 1.5 |
| Myristic | 8.9 ± 6.2 | ND | ND | ND |
| Myristoleic | 68.9 ± 2.4 | 76.3 ± 4.4 | 86.7 ± 4.2 | 79.0 ± 5.1 |
| Nervonic | 63.4 ± 10.3 | 63.1 ± 9.4 | 65.2 ± 6.8 | 69.3 ± 10.5 |
| Pentadecenoic | 5.7 ± 1.1 | 5.6 ± 1.1 | ND | 5.2 ± 0.7 |
| Triacontanoic | 27.3 ± 8.6 | 23.4 ± 6.1 | 20.3 ± 5.3 | 18.7 ± 4.4 |
